# Neuroimaging Parameters Are Not Associated With Chronic Post-stroke Fatigue in Young Stroke Patients

**DOI:** 10.3389/fneur.2022.831357

**Published:** 2022-04-28

**Authors:** Esther M. Boot, Sanne A. J. H. van de Camp, Noortje A. Maaijwee, Renate M. Arntz, Roy P. C. Kessels, Frank-Erik de Leeuw, Anil M. Tuladhar

**Affiliations:** ^1^Department of Neurology, Donders Institute for Brain, Cognition and Behavior, Radboud University Medical Centre, Nijmegen, Netherlands; ^2^Department of Neurology and Neurorehabilitation, Luzerner Kantonsspital Neurocentre, Luzern, Switzerland; ^3^Department of Neurology, Medisch Spectrum Twente, Enschede, Netherlands; ^4^Department of Psychology, Donders Institute for Brain, Cognition and Behavior, Radboud University, Nijmegen, Netherlands; ^5^Department of Medical Psychology and Radboudumc Alzheimer's Centre, Radboud University Medical Centre, Nijmegen, Netherlands; ^6^Vincent van Gogh Institute for Psychiatry, Venray, Netherlands

**Keywords:** young stroke, post-stroke fatigue, voxel-based lesion symptom mapping, brain network, graph theory

## Abstract

**Introduction:**

Post-stroke fatigue is frequently present in young adults, but its underlying mechanism is still unclear. The aim of the study was to investigate the association between lesion location, network efficiency and chronic post-stroke fatigue based on voxel-based lesion-symptom mapping and structural network connectivity analysis.

**Patients and Methods:**

One hundred and thirty five young patients, aged 18–50 years, with a first-ever transient ischemic attack or cerebral infarction from the Follow-Up of Transient ischemic attack and stroke patients and Unelucidated Risk factor Evaluation (FUTURE) study, underwent 1.5T MRI and were assessed for fatigue using the self-report Checklist Individual Strength. Stroke lesions were manually segmented, and structural network efficiency was calculated using the diffusion MRI-based brain networks and graph theory for each patient. Univariate and multivariate analyses was performed to study the associations between MRI parameters and chronic post-stroke fatigue. In addition, we used voxel-based lesion-symptom mapping to analyze the relationship between the lesion location and chronic post-stroke fatigue.

**Results:**

Mean age at index event was 39.0 years (SD ± 8.2), and mean follow-up duration was 11.0 years (SD ± 8.0). 50 patients (37%) had post-stroke fatigue. Voxel-based lesion-symptom mapping showed no significant relation between stroke lesions and the presence of chronic post-stroke fatigue. Furthermore, there were no significant associations between the lesion size or network efficiency, and the presence of chronic post-stroke fatigue.

**Discussion:**

We did not find any association between stroke characteristics (lesion location and size) and chronic post-stroke fatigue (CIS20-R), nor associations between structural brain network connectivity and post-stroke fatigue on the long term in young stroke patients.

## Introduction

Stroke in young adults (18–50 years) has a high socioeconomic impact due to amongst other the lifelong post-stroke consequences including post-stroke fatigue (PSF) (1). PSF affects at least 40% of young stroke survivors and may even be present several years after stroke. Moreover, PSF is related to poorer functional outcome and a higher mortality rate (2–4). Furthermore, patients consider PSF as one of the worst symptoms of stroke as it has a substantial impact on their quality of life (4).

Despite its importance, little is known about the underlying mechanisms of PSF. Lesion location may be an important determinant of PSF, but the associations between stroke topology and PSF remain unclear, as most studies did not show any associations between stroke side, location, and size and PSF (5–8). One of the major limitations is that most of these studies compared lesion sides and locations based on clinical diagnoses or on visible lesions on CT/MR imaging, which may not be sensitive for determining such associations. More precise methods, such as voxel-based lesion-symptom mapping (VLSM), may provide more insight into the particular brain regions that are associated with PSF in young stroke patients. VLSM is an increasingly utilized method to study the relationship between the location of stroke lesions and behavioral symptoms (9). This approach can be used to anatomically localize behavioral symptoms and has been used for investigating the structural basis of post-stroke depression (10) and post-stroke apathy (11).

An alternative explanation is that PSF is not primarily determined by the location of the stroke lesions, but rather by the brain networks affected by stroke. Recent studies have shown that cognitive and behavioral functions are distributed in the brain via several interconnected brain regions (12, 13). Network analysis studies the connections between the brain regions by investigating the organizational properties of the brain networks and has been used to investigate behavioral symptoms after stroke (13). Brain networks can be assessed with diffusion tensor imaging (DTI) and tractography. Using these techniques, it has been reported that lower global efficiency in an apathy-related subnetwork was an independent risk factor for post-stroke apathy (14). Recent study showed that functional connectivity of the fronto-striato-thalamic network predicted the response for chronic PSF treatment with modafinil (15). However, in this study structural connectivity that provide structural basis for the functional interactions between brain regions, was not examined. Therefore, it is not known whether disruption of the structural brain network, while looking beyond the effects of the stroke location, is also related to chronic PSF.

The aim of this study was to investigate the association between the location of stroke lesions, network efficiency, and chronic PSF by using VLSM and structural network connectivity analysis based on DTI. We hypothesized that lesion location is not an important determinant of chronic PSF, but that lower structural network efficiency is associated with chronic PSF in young ischemic stroke patients.

## Methods

### Patients

**Figure 1:**
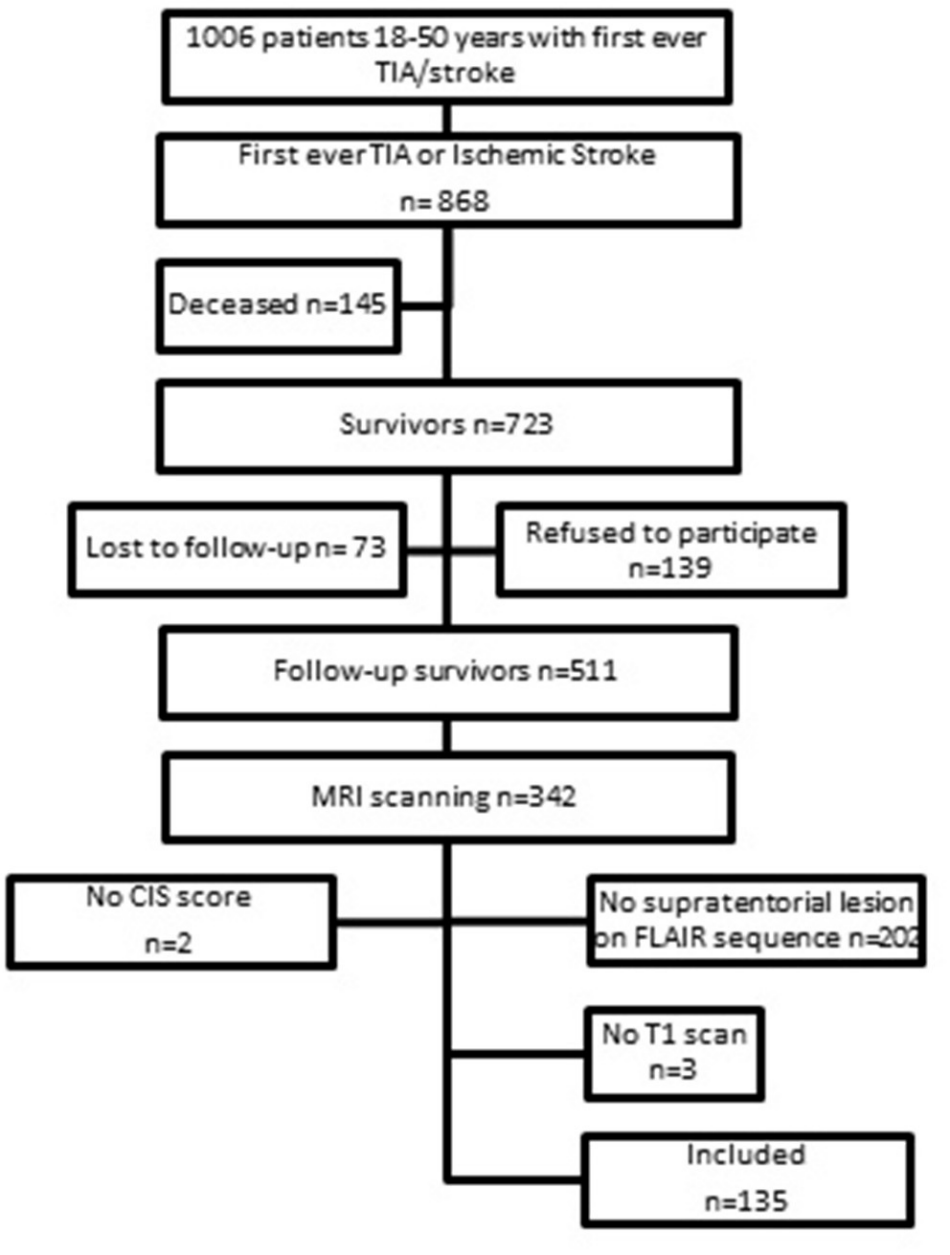
Flowchart of study population.

This study is part of the Follow-Up of Transient ischemic attack and stroke patients and Unelucidated Risk factor Evaluation (FUTURE) study, which is a prospective cohort study in which the etiologies and consequences of young stroke were investigated (16). Inclusion criteria were the presence of a transient ischemic attack (TIA), ischemic stroke of presumed arterial origin or intracerebral hemorrhage, which occurred between 1980 and 2010 in young adults aged 18–50 years who visited Radboud University Nijmegen Medical Centre. Although, there is no uniform age cut-off for defining “young stroke,” we used the age-limits 18–50 years (1, 17, 18). During the follow-up between 2009 and 2012 patients underwent neuropsychological screening and MRI scanning. The flowchart of the study population is shown in Figure 1. Only patients with the clinical diagnosis of an ischemic stroke or TIA and a supratentorial visible lesion on the MRI scan corresponding with the initial clinical diagnosis obtained at follow-up were included, which resulted in a total of 140 patients. We additionally excluded 5 patients (missing information on fatigue *n* = 2, and lack of T1 scans *n* = 3), yielding a total sample size of 135 patients for this study. The 135 patients included in this study had higher NIHSS-scores at admission [median 4 (IQR 2–10)] than patients excluded from the study (*n* = 376) [median 2 (IQR 0–4); *p* < 0.001] and more often had aphasia (24.6 vs. 10.4%, respectively, *p* < 0.001) based on NIHSS evaluated by the treating clinicians. Patients included in the study were also younger at the time of the index event compared to patients excluded from the study [mean 39.0 (SD 8.2) years vs. mean 40.7 (SD 7.7) years, *p* = 0.04]. No significant differences were found in clinical outcome at follow-up based on mRS.

### Post-stroke Fatigue

The presence of fatigue was assessed with the fatigue subscale of the self-report Checklist Individual Strength (CIS20-R) (19, 20), a questionnaire validated in stroke survivors (19, 21). Patients had to score 8 items on the level of fatigue over the past 2 weeks with scores ranging from 1 (yes, that is true) to 7 (no, that is not true). Examples of items are “I feel tired” and “Physically, I feel exhausted.” The total fatigue subscale score ranges from 8 to 56, with higher scores indicating more symptoms of fatigue. Severe fatigue is defined as a score >35 (2, 21, 22).

### Other Measurements

We defined depressive symptoms and anxiety using the Hospital Anxiety and Depression Scale (HADS) (23). The Dutch scoring system was used for the level of education, with 1 being less than primary school and 7 a university degree (16). The modified Rankin Score (mRS) was used to define functional outcome. MRS ranges from no symptoms (0 points) to death (6 points). We defined a good functional outcome as mRS <3 points (16).

### MRI Data Acquisition

Images were acquired on a 1.5T S Magneton Sonata scanner (Siemens Medical Solutions, Erlangen, Germany). The scanning protocol included (1) whole brain 3D T1 magnetization-prepared rapid gradient-echo (MPRAGE) sequence (TR/TE/TI 2730/2.95/1000 ms; flip angle 7°; voxel size 1.0 × 1.0 × 1.0 mm), (2) Fluid-attenuated inversion recovery (FLAIR) pulse sequences (TR/TE/TI 12220/85/2200 ms; voxel size 1.0 × 1.2 × 3.0 mm; slice gap 0.6 mm), (3) gradient echo susceptibility weighted imaging sequence (TR/TE 49/40 ms; voxel size 0.8 × 0.7 × 1.0 mm), (4) DTI (TR/TE 9100/98 ms; voxel size 2.2 × 2.2 × 2.2 mm; 7 unweighted scans, 61 diffusion-weighted scans, with non-co-linear orientation of the diffusion-weighting gradient, and b value 1,000 s/mm^2^).

### Lesion Segmentation

All chronic stroke lesions were manually segmented on the FLAIR sequence using ITK-SNAP by a trained investigator (SC). All lesion borders were checked by an experienced clinician (EB). We manually segmented stroke lesion and the adjacent glial scars. After lesion segmentation, we registered the lesion masks non-linearly to a standard space. To this end, the Functional MRI of the Brain non-linear registration tool (FNIRT) was used to non-linearly register the skull-stripped T1-weighted images to Montreal Neurological Institute (MNI) 152 template. Next, we registered both the masks of the stroke lesions and the FLAIR images to the standard space using the transformation matrix of T1-weighted images to standard space after linearly registering to T1-weighted image using Functional MRI of the brain linear image registration tool (FLIRT). We used FSL 5.0.5 tools (24).

### Structural Network

We defined network nodes (i.e., brain regions) using the Automated Anatomical Labeling (AAL) template (25), which resulted in 90 regions (45 per hemisphere), excluding the cerebellum. The AAL image was registered to each participant's diffusion image space by using the earlier obtained transformation matrixes.

We defined our network edges [i.e., white matter (WM) connections] by the following procedures. First, we used “PATCH” on the raw diffusion data to correct for cardiac and head motion artifacts and for eddy currents (26). Second, we calculated the diffusion tensor and mean fractional anisotropy (FA) using DTIFit from FSL. Third, we used fiber assignment by continuous tracking (FACT) to generate the WM tracks for the whole brain for each participant. The tracking algorithm started at the center of the voxels with fractional anisotropy >0.2 and ended when the fiber tracks left the brain mask, encountered voxels with fractional anisotropy <0.2 or when the turning angle exceeded 60°. Two regions were considered connected if the endpoints of the reconstructed streamline lay within both regions. Fourth, the weight of the connection (e.g., the connection strength) was defined as mean fractional anisotropy (FA) of the reconstructed streamlines multiplied by the number of reconstructed streamlines connecting two regions (27). Finally, we corrected for the differences in the AAL regions size and the differences in brain size (28), resulting in an undirected, weighted 90 × 90 matrix for each participant.

### Graph Theory Analysis

We assessed graph-theoretical network measures using the Brain Connectivity Toolbox (29). We calculated 5 different network measures: (1) network strength, (2) density, (3) global efficiency, (4) local efficiency and (5) nodal efficiency. Degree defines the number of neighbors of a node. Network strength is weighted variant of degree and represents the sum of all neighboring link weights. Density represents the fraction of present connections to possible connections. Global efficiency is the average inverse shortest path length in the network. Local efficiency is global efficiency computed on node neighborhoods. Nodal efficiency is the mean of the inverse of the shortest path length between the node and all other nodes in the network. Furthermore, we performed a *post-hoc* analysis in which the network measures were calculated from the unaffected hemisphere.

### Statistical Analysis

We analyzed group differences between fatigued and non-fatigued patients for demographic, clinical variables using χ^2^-tests or Fisher's exact tests for categorical variables and *t*-test for the continuous variables, where appropriate. To investigate the relationship between lesion location and the presence of chronic PSF, we used voxel-based lesion mapping (VLSM) (9) implemented in the non-parametric mapping (NPM) software of MRIcron. In the univariate analysis, we used the Brunner and Munzel test (30) and a *t*-test with 1,000 permutations with a p-threshold of 0.05, corrected for multiple comparisons using Benjamini-Hochberg (BH) false discovery rate (FDR) correction (31). Next, we performed a multivariate analysis by using NiiStat in Matlab (https://github.com/neurolabusc/NiiStat). We also analyzed group differences for imaging variables, including lesion location, lesion volume and network measures. For significant associations, we additionally adjusted for possible confounders; age at follow-up, education, depressive symptoms, anxiety, and mRS score during follow-up. In case of multiple comparisons, we used the Bonferroni correction to adjust for multiple comparisons. Furthermore, we investigated whether a specific subnetwork for chronic PSF could be found by analyzing group differences for nodal efficiency of each brain region, by using unpaired *t*-tests, corrected for multiple comparisons using BH FDR correction. All statistical analyses were carried out in the statistical software IBM SPSS Statistics 26 and Matlab R2021a.

## Results

### Study Population

We included 135 patients with a mean age of 39.0 years (SD ± 8.2) at index event. Mean follow-up duration was 11.0 years (SD ± 8.0). The mean CIS20-R-score was 30.3 (SD ± 13.3) and 50 patients (37%) patient fulfilled the criteria of severe PSF. Depression and anxiety were significantly more present in the fatigued group than in the non-fatigued group. The demographic and clinical variables are shown in Table 1.

**Table 1:** Demographic and clinical variables of fatigued and non-fatigue young stroke patients.

	**Whole group (*n* = 135)**	**Fatigue (*n* = 50)**	**Non-fatigue (*n* = 85)**	**P-value**
Age at index event, mean (sd)	39.0 (8.2)	39.5 (8.4)	38.8 (8.1)	0.62
Follow-up duration, mean (sd)	11.01 (8.0)	9.26 (8.0)	12.05 (7.8)	0.05
Male, *n* (%)	60 (44.4)	18 (36.0)	42 (49.4)	0.13
Education level (range)	4.71 (6)	4.58 (6)	4.79 (5)	0.35
Stroke type, *n* (%)
• TIA	12 (8.9)	4 (8.0)	8 (9.4)	0.78
• Ischemic stroke	123 (91.1)	46 (92.0)	77 (90.6)	
NIHSS at admission, mean (sd)	6 (5)	6 (5)	6 (5)	0.79
CIS20-R fatigue score at follow-up, mean (sd)	30.3 (13.3)	44.8 (6.3)	21.8 (7.9)	**<0.001**
Recurrent stroke, *n* (%)	24 (17.8)	13 (26.0)	11 (12.9)	0.05
mRS score at follow-up, mean (sd)	1.24 (0.9)	1.50 (1.02)	1.09 (0.7)	**0.01**
Depression at follow-up, *n* (%)	20 (14.8)	16 (32.0)	4 (4.7)	**<0.001**
Anxiety at follow-up, *n* (%)	25 (18.5)	21 (42.0)	4 (4.7)	**<0.001**

*sd, standard deviation; TIA, transient ischemic attack; NIHSS, National Institutes of Health Stroke Scale; CIS, Checklist Individual Strength; mRS, modified Rankin Scale. Bold indicates significant P-values (P < 0.05)*.

### MRI Correlates of Fatigue in Young Stroke

#### Lesion Volume and Lesion Side

The results of the univariate and multivariate analyses are shown in Table 2. The univariate analysis showed a higher risk of chronic fatigue after left hemisphere stroke compared to right hemisphere stroke, which was not significant, after controlling for potential confounders (lesion size, age at follow-up, sex, educational level, depression, anxiety, and mRS) (*p* = 0.06).

**Table 2:** MRI variables of fatigued and no fatigue young stroke patients.

**Variable**	**Whole group (*n* = 135)**	**Fatigue (*n* = 50)**	**Non fatigue (*n* = 85)**	***p*-value**	**OR^∧^(95% CI)**	**P-value^∧^**
Lesion side, *n* (%)						
• Left	53 (39.3)	27 (54.0)	26 (30.6)	Ref	Ref	Ref
• Right	63 (46.7)	18 (36.0)	45 (52.9)	0.05^a^	0.34 (0.13-0.90)	0.06*^a^*
• Bilateral	19 (14.1)	5 (10.0)	14 (16.5)	0.21^a^	0.48 (0.12-1.98)	0.75*^a^*
Lesion size (ml), mean (sd)	46.26 (55.99)	36.88 (52.80)	51.42 (57.34)	0.18		
Density, mean (sd)	0.08 (0.01)^*^	0.08 (0.01)^**^	0.08 (0.02)^***^	0.46		
Strength, mean (sd)	4.05 (0.84)^*^	4.18 (0.84)^**^	3.98 (0.84)^***^	0.23		
Global efficiency, mean (sd)	1.67 (0.62)^*^	1.77 (0.63)^**^	1.62 (0.61)^***^	0.21		
Local efficiency, mean (sd)	1.63 (0.52)^*^	1.67 (0.51)^**^	1.61 (0.53)^***^	0.52		

* *n = 121, patients without DTI were excluded*,

** *n = 43, patients without DTI were excluded*,

*** *n = 78, patients without DTI were excluded*.

∧ *Adjusted for potential confounders (lesion size, age at follow-up, sex, educational level, depression, anxiety, and mRS)*.

a *P-values are Bonferroni corrected*.

#### Voxel-Based Lesion Mapping

**Figure 2:**
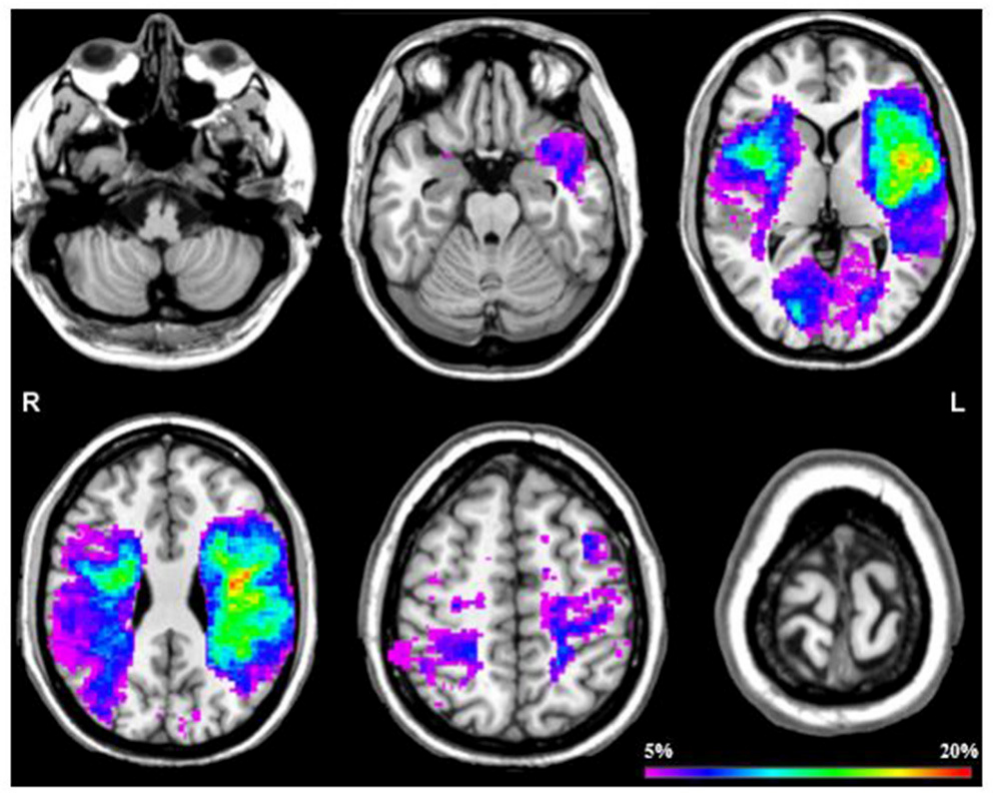
Probability map of all stroke lesions. This probability map displays a overlap across all stroke patients by 6 slices at *z* = 25, *z* = 50, *z* = 75, *z* = 100, *z* = 125 en *z* = 150. The color scale saturates at 20% which means that in 20% of the patients that specific area was damaged.

Figure 2 shows an overlap image of all stroke lesions. The univariate VLSM analysis showed no significant difference in lesion location between the fatigued group compared to the non-fatigued group after correcting for multiple testing (FDR correction *q* < 0.05). This was further confirmed by the multivariate VLSM, while correcting for the potential confounders.

#### Brain Networks

Whole brain network strength, network density, global efficiency and local efficiency were not significantly associated with the presence of chronic PSF (Table 2). In the *post-hoc* analysis, network measures from the unaffected hemisphere were also not significantly associated with the presence of chronic PSF. Furthermore, we found no significant differences in the nodal efficiency of each brain region between the fatigued group compared to the non-fatigued group after correcting for multiple testing.

## Discussion

In this study, we found no association between stroke characteristics (e.g., lesion location or lesion volume) and chronic PSF (based on CIS20-R), nor between structural network connectivity and chronic PSF in young stroke patients. These findings indicate that structural brain measures (characteristics of stroke lesions and structural brain networks) are not important determinants of PSF on the long term in young stroke patients.

We performed VLSM to investigate the relationship between stroke location and chronic PSF in a more robust manner than previous studies. Our analysis did not show any differences in (damaged) brain structures in young stroke patients with chronic PSF compared to patients without PSF. Our findings are comparable to previous studies showing no associations between either visible stroke lesions or lesions detected with VLSM and PSF in patients during the chronic stage of stroke (32–36). This is in contrast with studies showing significant association between lesion location (e.g., infratentorial and deep circulation) and PSF (21, 37–41) that included patients in the acute phase of the stroke (21, 37–40, 42). There are several factors that may explain the lack of association between brain measures and PSF. First, fatigue is a multidimensional entity in which predisposing factors, coping skills, stroke outcome, and mood disorders (19, 43) may play an important role, with lesion location adding little to the explained variance, especially on the long term. Second, although stroke lesions are considered permanent, their relation to behavioral function may change over time due to neuroplasticity and recovery. These changes may explain the lack of an association between the stroke lesion and chronic fatigue more than a decade after the initial event. Third, stroke lesions may result in remote damage to structurally intact regions, leading to disruption of the structural networks. The stroke lesion itself might not truly represent the brain changes that might be involved in chronic PSF.

However, we did not find associations between structural network connectivity and chronic PSF. These results are in line with findings in the general stroke population showing no associations between chronic PSF and structural network measures or lesion characteristics (44). A recent study showed that the functional connectivity of the fronto-striato-thalamic network could predict the response for PSF treatment with modafinil (15). This suggests that specific subnetworks may be associated with PSF. In contrast, we did not find any differences in nodal efficiency of the brain regions between fatigued and non-fatigued patients in the chronic phase of stroke. This implies that a specific subnetwork is not involved in PSF in our study. An explanation, as we stated earlier, is that PSF is multifactorial (43) and other factors, for example mood disorders, are more important contributors.

Consistent with previous studies, we found that chronic PSF was associated with depressive symptoms and anxiety (21, 43, 45, 46). One explanation could be that fatigue may be the result of symptoms of depression and anxiety (21, 35). However, it has been shown that PSF and depression are two different clinical entities that can be dissociated from each other, since not all patients with PSF has depressive symptoms or anxiety and vice versa (37, 45). Furthermore, in this study, we used the CIS20-R fatigue score to assess the level of fatigue. Though validated to assess fatigue in stroke, we cannot distinguish between the different subtypes of fatigue. Since depression and anxiety are more likely correlated with psychological or mental fatigue and less likely with physical fatigue (21, 37), future research should investigate the association between depressive symptoms and anxiety using instruments that enable to disentangle different subtypes of fatigue.

Strengths of our study include the large sample size of young stroke patients, the long follow-up duration of 11 years and the single-centre design. Furthermore, to the best of our knowledge, this is the first study to investigate the relationship between structural brain measures and chronic PSF using VLSM and structural network analysis in young stroke patients who are free from other neurological disorders.

However, there are several limitations to be mentioned. First, we do not have information about predisposing factors (43). In a conceptual model (43), predisposing factors may be involved in the occurrence of PSF, including vulnerability to stress (e.g., personality, coping style and illness perceptions), pre-stroke fatigue (6, 47) or pre-stroke depressive symptoms (41). Second, early effects of stroke, such as stroke lesions (21, 37–40, 42), stroke-related inflammatory and neuroendocrine changes, and attentional-executive impairments have been described as triggers for early fatigue. In this study, there is no information available about early fatigue. Also, we have no information on other stroke-related factors that might change over time and contribute to the presence of chronic PSF. Third, we used the fatigue cut-off score of 35 (indicating severe fatigue) as used in previous post-stroke fatigue studies (2, 21). We also performed additional analyses in which an alternative fatigue cutoff was considered to include patients with mild and moderate fatigue. However, the analyses with a cutoff score of >26 remained similar, showing no differences between patients with fatigue and without fatigue in terms of MRI parameters. Fourth, in this study, we included patients who had a mean follow-up duration of 11.0 years. In those years, several events (e.g., socio-economic and other life events, comorbidities and/or newly started medication) may have either attenuated, increased or resolved their fatigue. Fifth, the generalizability of our study might be affected because we were only able to use a subset of the FUTURE study cohort. The included patients were significantly older than the excluded patients, however the absolute difference in mean was 1 year. There is conflicting evidence about the association between age and the risk of post-stroke fatigue, as some studies showed a relation between increasing age and the risk of fatigue, while others showed no relation or even opposite relation (21, 48). Although the role of age as a factor for the risk of post-stroke fatigue still needs to be elucidated, it is unlikely that the lack of association between MRI-parameters and post-stroke fatigue in our study is influenced by age. Also, the patients included in this study were more affected at admission than the patients excluded from the study and there were no significant differences among clinical outcome at follow-up. At admission, the included patients more often had aphasia than the excluded patients. Although the evaluation of post-stroke fatigue in patients with aphasia can be challenging, there is some evidence that patients with language impairment more often have fatigue than patients without language impairment (49). In our study, however, no information was obtained during the follow-up regarding their language abilities. Therefore, we were unable to investigate the impact of aphasia at follow-up on (the report of) post-stroke fatigue. Furthermore, the prevalence of severe fatigue in this study (37%) was in line with the prevalence of severe fatigue in all ischemic stroke patients of the FUTURE study (2). Therefore, our results should be generalizable to the whole study population.

## Conclusion

Chronic PSF in young stroke patients seems to be a multi-dimensional and multifactorial symptom, which is not associated with stroke characteristics and structural network measures. Future studies are needed to better understand the mechanisms of PSF, focusing more on the role of predisposing factors of fatigue and its different subtypes, and the role of stroke lesions in the acute phase.

## Data Availability Statement

The raw data supporting the conclusions of this article will be made available by the authors, without undue reservation.

## Ethics Statement

The studies involving human participants were reviewed and approved by Medical Ethics Committee Region Arnhem-Nijmegen. The patients/participants provided their written informed consent to participate in this study.

## Author Contributions

SC, EB, and AT were involved in data analysis and wrote the first draft of the manuscript. All authors reviewed and edited the manuscript and approved the final version of the manuscript.

## Funding

AT was a junior staff member of the Dutch Heart Foundation (Grant No. 2016T044). F-EL has received the Innovational Research Incentive grant (016-126-351) and the Clinical established investigator Dutch Heart Foundation grant (2014 T060).

## Conflict of Interest

The authors declare that the research was conducted in the absence of any commercial or financial relationships that could be construed as a potential conflict of interest.

## Publisher's Note

All claims expressed in this article are solely those of the authors and do not necessarily represent those of their affiliated organizations, or those of the publisher, the editors and the reviewers. Any product that may be evaluated in this article, or claim that may be made by its manufacturer, is not guaranteed or endorsed by the publisher.
